# Durability of Blended Cements Made with Reactive Aggregates

**DOI:** 10.3390/ma14112948

**Published:** 2021-05-29

**Authors:** Esperanza Menéndez, Miguel Ángel Sanjuán, Ricardo García-Roves, Cristina Argiz, Hairon Recino

**Affiliations:** 1 The Eduardo Torroja Institute for Construction Science (Spanish National Research Council, CSIC), C/Serrano Galvache, 4, 28033 Madrid, Spain; emm@ietcc.csic.es (E.M.); r.gloza@alumnos.upm.es (R.G.-R.); h.recino@ietcc.csic.es (H.R.); 2 Civil Engineering School, Technical University of Madrid (UPM), C/Profesor Aranguren, 3, Ciudad Universitaria, 28040 Madrid, Spain; cg.argiz@upm.es

**Keywords:** building materials, durability, microstructure, alkali–aggregate reaction (AAR) mitigation, natural pozzolan, coal fly ash, silica fume, blast-furnace slag, circular economy, sustainability

## Abstract

**Featured Application:**

**In this paper, blended cements are proposed as an effective means of meeting the needs of mitigating climatic change. This proposal is a two-pronged strategy, i.e., durable and sustainable. The pozzolanic reaction of four binders is assessed, which is related to an alkali–silica reaction (ASR). Thanks to the findings made here, mix-design optimization can be performed.**

**Abstract:**

Alkali–silica reaction (ASR) is a swelling reaction that occurs in concrete structures over time between the reactive amorphous siliceous aggregate particles and the hydroxyl ions of the hardened concrete pore solution. The aim of this paper is to assess the effect of pozzolanic Portland cements on the alkali–silica reaction (ASR) evaluated from two different points of view: (i) alkali-silica reaction (ASR) abatement and (ii) climatic change mitigation by clinker reduction, i.e., by depleting its emissions. Open porosity, SEM microscopy, compressive strength and ASR-expansion measurements were performed in mortars made with silica fume, siliceous coal fly ash, natural pozzolan and blast-furnace slag. The main contributions are as follows: (i) the higher the content of reactive silica in the pozzolanic material, the greater the ASR inhibition level; (ii) silica fume and coal fly ash are the best Portland cement constituents for ASR mitigation.

## 1. Introduction

In response to meeting the global warming target of 1.5 °C, signed in the Paris Agreement, an area of paramount concern regarding climatic change mitigation is that of reaching the goal of carbon neutrality [1]. Furthermore, another key aspect of sustainable development is circular economy. Comprehensive national and international development strategies should be implemented for mitigating climate change and promoting circular economy, as do the roadmap to a resource efficient Europe [2] and the circular economy package in Europe [3], which include regulations for recycling 65% of global municipal waste by 2035, in line with the European Green Deal targets [1,3]. These legislative initiatives will aim to make products fit for a climate-neutral circular economy that is resource efficient. It is expected that the performance of industry in sustainability progressively becomes the norm. 

Accordingly, carbon neutrality in the cement and concrete sector is expected to be achieved through the entire clinker, cement, and concrete value stream by 2050 [4]. Blended cement production can be considered as a key lever to achieve the net zero emissions future [4]. Industrial wastes and by-products used in such cements should provide a durable and safe final product.

Pozzolans are natural or artificial materials that consist mainly of silica and alumina and are able to combine with portlandite, Ca(OH)_2_, in the presence of water to produce calcium silicate hydrate (or C-S-H gel) exhibiting a binding character.

Pozzolanic constituents in Portland cements afford some good characteristics to the cement-based materials made with them. Firstly, they improve the mechanical strength of mortars and concretes. Secondly, they are capable of binding calcium hydroxide in the presence of water, which is a weak Portland cement hydration product. In addition, a calcium silicate with a low Ca/Si ratio is formed, with good durable and cementing properties. Accordingly, materials that exhibit pozzolanic reactivity can lower hydration heat, improve the durability, and increase the service life of concrete structures exposed to marine and other aggressive environments [5]. Then, the addition of pozzolans to Portland cement results in increased mechanical strength when compared to the plain Portland cement because of the pozzolanic reaction products formation and filler effect of the finer particles. They provide a denser and more uniform cement paste. 

Furthermore, pozzolanic constituents partially replace the clinker in Portland cements [6], lowering the global carbon dioxide emissions. As the cement industry emits about 7.4% of the global emissions (2.9 Gtons) [7], the reduction in the clinker factor, i.e., clinker to cement ratio, is a significant lever for decarbonization. Consequently, an increase in the use of blended cements in concrete is expected worldwide in order to meet the target of maintaining the global temperature increase below 1.5 °C by the end of this century [4].

Aggregates are inert granular materials, in most of the cases, that are an essential ingredient in concrete because they fill between the seventy-five and eighty-five percent of the concrete volume. In some cases, they react with the alkaline pore solution of the concrete. Accordingly, the alkali–silica reaction (ASR) occurs in concrete [8], which is a deleterious swelling reaction between the highly alkaline pore solution [9] and the amorphous reactive silica present in some common aggregates [10], as it draws water from the surroundings. This reaction is a two-step process: (i) alkalis react with the reactive silica present in the aggregate producing alkali-silica gel; (ii) such alkali–silica gel imbibes water and swell producing expansion. However, some alkali–silica gels expand little or not at all. Therefore, the presence of alkali–silica gel does not always indicate deleterious alkali–silica reaction (ASR). By contrast, high-swelling gel may produce cracking and loss of durability and mechanical strength in concrete structures.

Common sources of alkali are as follows: Portland cement paste, aggregates, and surrounding area. Alkali ions react with water, leading to the alkali hydroxide formation [11]. Although silica in the form of quartz is chemically inert, the poorly crystalline silica present in some aggregates reacts with the alkaline pore solution to produce amorphous hydrous silica. Thus, alkali–silica reaction (ASR) proceeds according to several steps [11]. In the first stage, the bonds between different siloxane groups (Si-O-Si) are broken and new bonds formed, so creating new alkali silicate gel and silicic acid (silanol bonds). Later, alkali silicate gel reacts with the calcium ions present in the cement paste and forms alkali–calcium silicate hydrate gel. In the second stage, alkali–calcium silicate hydrate gel imbibes and swell. Therefore, the internal swelling pressure increases promoting crack formation and crack growth in concrete.

One of the best options to mitigate the alkali–silica reaction (ASR) is the blended cement usage when pozzolanic materials are employed [12,13]. The good ASR mitigation performance can be justified by the alkali dilution effect when the clinker content is reduced in the blended cement. In addition, the pozzolanic C–S–H gel provides new nucleation sites for absorption of the alkalis and promotes the densification the cement paste. Furthermore, the type of the addition and content used in the Portland cement is a key factor regarding the mitigation effectiveness. They can control alkali–silica reaction (ASR) because they bind alkalis and, therefore, alkali availability for ASR is reduced [14]. Consequently, the chemical composition of the pozzolanic materials and the final blended cement will play a key role in their effectiveness on the alkali–silica reaction (ASR) mitigation. Pozzolanic materials lower the alkali–hydroxides concentration and pH in the pore solution of mortars and concretes [15,16]. Accordingly, the higher the level of substitution, the lower the alkali–hydroxide concentration in the pore solution.

It has been reported that a low content of silica fume (5–10%) in cement-based materials showed a lowering of hydroxyl ion concentration in the pore solution at 28 days, but it raises at three months [15]. 

By contrast, blast-furnace slag cements and coal fly ash cements do not release alkalis after a long period of time, probably due to the incorporation of the Al in C–S–H gel, which improves the alkali binding capacity [16] and limits the further alkali availability for reaction with aggregates [14]. This is explained by the C–A–S–H gel formation which has a high alkali-binding capacity [16]. The role of the alumina on the alkali-binding capacity must be further studied.

Another aspect that should be considered is how the pozzolanic additions react with regard to the alkali–silica reaction (ASR). Both types of chemical reactions are quite similar. Reactive silica from the aggregate or from the pozzolanic material reacts with alkali–hydroxides forming alkali–silica gel. Then, in the Portland cement with pozzolanic additions, calcium exchanges for sodium and potassium in the alkali–silica gel and a new C–S–H gel with a lower Ca/Si ratio than that of the Portland cement pastes is formed. By contrast, the absence of expansion in the pozzolanic reaction is the main difference with the alkali–silica reaction (ASR), which is attributed to the high fineness of the reactants. This in turn enables the alkali-silica reaction (ASR) gel generated in the pozzolanic reaction to be dispersed throughout the cement paste. On the contrary, local deposits of alkali–silica gel are formed on the coarser aggregates [9].

In a nutshell, the effectiveness of silica fume, metakaolin and siliceous fly ash in mitigating ASR is based on [14] (i) dilution effect; (ii) additional pozzolanic C–S–H gel with lower Ca/Si ratio formation with new nucleation sites for alkali’s absorption; (iii) cement paste densification and reduction in permeability (capillary pores and interfacial transition zone (ITZ) filling with the pozzolanic C–S–H gel) [15].

The main objective of this research is to assess ASR mitigation by using blended cements made with four pozzolanic materials and a highly reactive aggregate. Considering this intention, mechanical, microscopical and ASR-induced expansion were outlined and performed. 

## 2. Materials and Methods

### 2.1. Materials

A common Portland cement CEM I 42.5N (EN 197-1 [17]), supplied by the Hontoria cement plant (Palencia, Spain) belonging to The Cementos Portland Valderrivas Group, was utilized as reference cement and to prepare some blended cements containing natural pozzolan, P, siliceous coal fly ash, V, silica fume, D, or ground granulated blast-furnace slag, S. 

Table 1 shows the blended cements mix design to be used for ASR testing. The percentage of pozzolanic material selected for Portland cement replacement in the binder adequately represent the main types of commercial cements. 

The percentage of the additions in the blended cements to be used for porosity and mechanical strength testing was 10%, 20%, 30%, 40% and 50% for all the pozzolanic materials. In addition, 5% was also used for siliceous coal fly ash, silica fume, and ground granulated blast-furnace slag. Furthermore, 3%, 7% and 15% of silica fume was also tested.

The siliceous fine aggregate (4–6 mm) employed in this study was provided by El Aljibe quarry (Almonacid, Toledo, Spain). This is an alkali-bearing reactive aggregate according to UNE 146,508 [18]. Distilled water was used for all the mortar mixes.

### 2.2. Chemical and Mineralogical Characterization of the Materials

Chemical compositions of the Portland cement, CEM I, siliceous coal fly ash, V, ground granulated blast-furnace slag, S, silica fume, D, natural pozzolan, P, and reactive aggregate are given in Table 2. Chemical analyses were performed by X-ray Fluorescence (XRF) by using a S8 Tigger 4 kW model instrument (Bruker, Billerica, MA, USA), whereas chloride ion concentration and loss on ignition (LOI) were measured in accordance with the European standard EN 196-2 [19].

**Figure 1:**
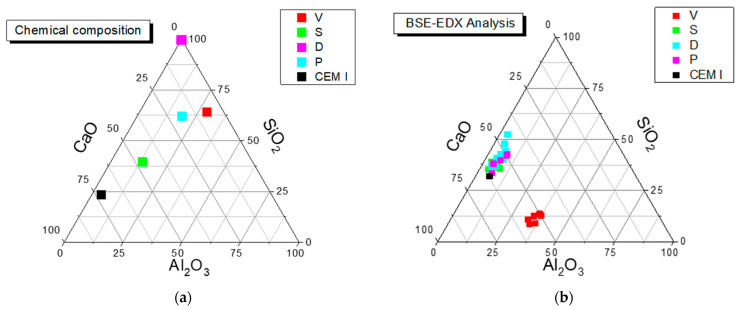
Ternary plot showing the main chemical composition for: (**a**) pozzolanic additions and CEM I; (**b**) mortar mixes made with four pozzolanic additions and the reference cement (CEM I). It accurately graphically depicts the values of the three variables CaO, SiO_2_ and Al_2_O_3_, which sum up to exactly 100%.

Figure 1 depicts a ternary plot representing the main chemical composition of the four pozzolanic additions and the reference cement (CEM I) and the mortar mixtures made with them. Such chemical composition was determined with scanning electron microscopy energy-dispersive X-ray analysis (SEM-EDX). 

Figure 1 accurately graphically depicts the values of the three variables CaO, SiO_2_ and Al_2_O_3_, which sum up to exactly 100%. It is noticeable that coal fly ash mortars present quite a different chemical composition to the rest of the mortars.

The highest Na_2_O_eq_ content is found in the reactive aggregate (5.89%), coal fly ash (4.56%) and silica fume (3.22%). These values are much higher than those found in Portland cement, ground granulated blast-furnace slag and natural pozzolan. Accordingly, the effect of alkali release from aggregates during the alkali–silica reaction (ASR) should be considered [20]. Furthermore, alkalis have a significant effect on the mechanical strength gain and flowability of mortars and concretes.

### 2.3. Particle Size Disribution

The particle-size distribution (PSD) of the binder materials was measured using a laser scattering technique to define the relative amount by mass of particles present according to size. The major illumination source in a Beckman–Coulter LS-13 320 for particle size distribution (PSD) measurement is a 5 mW laser diode with a wavelength of 750 nm. This equipment provides PSD in the form of 93 bins in the range of 0.38–2000 μm of dimeter, i.e., 19 bins (0.38–2 μm) plus 74 bins (2–2000 μm). 

Because the analyzer required a small subsample lower than 5 g for the particle-size distribution (PSD) measurement, each sample was blended several times before testing to minimize the variability of the results. Later, each sample was subjected to 60 s of optical measurement.

**Figure 2:**
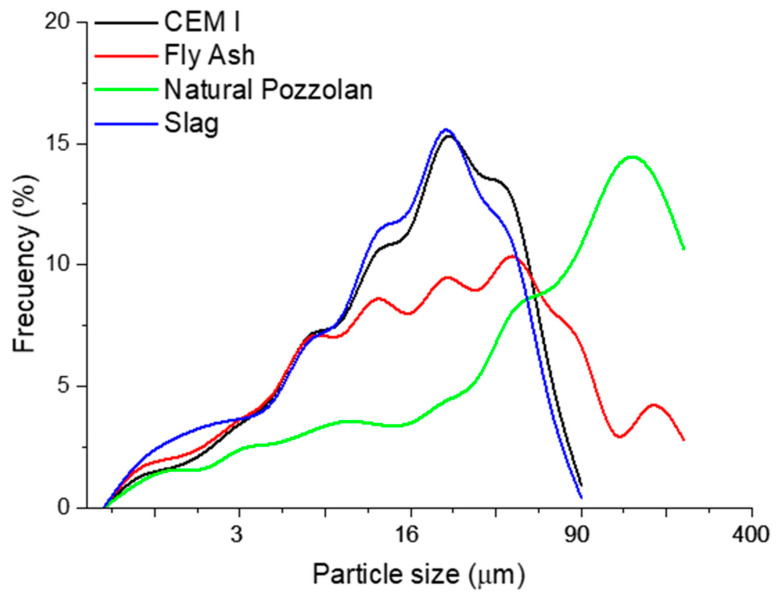
Particle size distribution curve for the natural pozzolan, siliceous coal fly ash, ground granulated blast-furnace slag and the reference cement (CEM I).

Figure 2 shows the Particle Size Distribution Curve for the natural pozzolan, siliceous coal fly ash, ground granulated blast-furnace slag and the reference cement (CEM I). Silica fume particles have an average diameter between 0.1 and 0.2 μm, which is roughly 1/100 of the mean Portland cement particle. Therefore, the Beckman–Coulter LS-13 320 for particle size distribution (PSD) measurement cannot provide an accurate particle size distribution curve. The PSD is one of the most basic and important properties of pozzolanic materials. Ground granulated blast-furnace slag presents quite a similar particle size distribution curve to the reference cement (CEM I), whereas the results of grain-size analysis for the coal fly ash exhibit a wider area and higher mean grain size. However, there are great differences between the natural pozzolans and the rest of the materials, which present a coarser average grain size. Consequently, they have a smaller surface area and reactivity, which is dependent upon particle size and surface area.

### 2.4. Mechanical Strength and Open Porosity

Compressive and flexural strength determination was performed at 2, 7 and 28 days according to the mechanical strength procedure given in the European standard EN 196–1 [21]. 

Open porosity of the mortar samples cured for 28 days of wet curing was assessed according to the method defined in the Spanish standard UNE 83,980 [22]. This parameter was calculated using Equation (1), where m_1_ is the sample weight after drying at 110 °C ± 5 °C for 24 h, m_2_ is the sample weight after vacuum conditions (2.0 ± 0.7 kPa ≅ 15 ± 5 mmHg) and m_3_ is the apparent weight (i.e., hydrostatic weight).

(1) $$Open\;porosity,\;\%=\frac{m_{2}-m_{1}}{m_{2}-m_{3}}\cdot 100$$

### 2.5. Alkali–Aggregate Reactivity Test Method (Expansion of Mortar-Bar Method)

The alkali–aggregate test method (blended cement mortar-bar method) used in this investigation is given in the Spanish standard UNE 146508 [18]. The aggregates were washed, dried at 105 °C ± 5 °C, crushed and, finally, sieved into five fractions which ranged from 0.160 mm to 5 mm according to UNE 146508 [18]. The potential reactivity of the aggregate in the mortars was assessed in three mortar prisms (2.5 × 2.5 × 28.5 cm^3^) for each cement. Mortars were elaborated by mixing 400 g of cement and 900 g of aggregate with a water/cement ratio of 0.47 (by weight), and the aggregate/cement ratio of 2.25 (by weight). Some molds with a stainless-steel gauge stud located in both ends of the prism were used. The specimens were demolded after 24 h and submerged in water at 80 °C ± 1 °C for 24 h. 

Once the specimens were taken out from the reservoir tanks, the initial readings were measured. Afterwards, the prisms were stored in the 1 N NaOH solution at 80 °C ± 1 °C in containers at 80 °C ± 1 °C for 14 days. Further expansion readings were recorded from 2 to 14 days prescribed by the UNE 146508 standard [18]. Furthermore, additional measurements were taken until 90 days. 

Mortar-bar expansion for each exposure time was calculated using Equation (2), and the average readings of the three prisms was considered. Aggregates were assessed with the 14-day expansion results. Accordingly, a mortar bar expansion of lower than 0.10% indicates a non-reactive aggregate [18]. In addition, an expansion over 0.20% at 28 days shows a potentially reactive aggregate.

(2) $$\mathrm{Mortar}\;\mathrm{bar}\;\mathrm{expansion}\;\left(\%\right)=\frac{\left(L_{n}\;–{\;L}_{0}\right)}{L_{c}\;}\times 100$$

where L_0_ is the initial length, L*_n_* is the testing time length and L*_c_* is the calibration length (L*_c_* = 254 mm). 

## 3. Results and Discussion

### 3.1. Open Porosity

**Figure 3:**
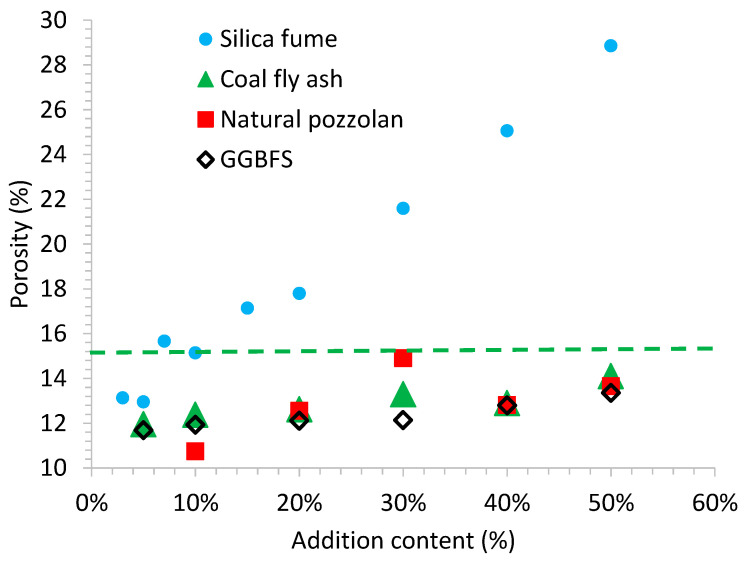
Mortar’s open porosity. The effect of the four additions (natural pozzolan, P, siliceous coal fly ash, V, silica fume, D, and ground granulated blast-furnace slag, GGBFS) is compared with the reference mortar, shown by green dashed line.

Figure 3 illustrates the mortar’s open porosity measured after 28 days of wet curing. The reference mortar without additions presented an open porosity of 15.25%, while most of the porosities in the mortars made with the blended cements were found between 12% and 14%, independently of the type of constituent. This reduction can be explained by the pozzolanic activity of the four additions. However, silica fume mortars followed a clear upward trend of 7%, i.e., the higher the silica fume content is, the higher the recorded open porosity. This linear relationship can be justified as follows: a 7% substitution of Portland cement by silica fume provided a similar open porosity to the rest of additions. Silica fume particles, afforded nucleation sites for Portland cement, hydrate growth. Accordingly, the pozzolanic reaction took over and increased C-S-H gel formation in the capillary pores. By contrast, when the silica fume content increases from 15 to 50%, the open porosity increases since, at a subsequent phase, the Portland cement hydration rate slows and less Ca(OH)_2_ is formed. Given that the pozzolanic reaction is controlled by the calcium hydroxide formation, it depends on calcium hydroxide availability [23]. Consequently, the excess silica fume acts purely as an inert filler.

### 3.2. Alkalies in the Mortars Made with Blended Cements

Table 3 shows the alkali content, which refers to the content of Na_2_O and K_2_O in the mortars made with blended cements. Because the Na_2_O_eq_ content (calculated by Na_2_O + 0.658 K_2_O) in the coal fly ash (4.56%) and silica fume (3.22%) is high, mortars made with these additions showed the higher values. In addition, the reactive aggregate does actually have a higher value (5.89%). In contrast, the Portland cement showed the lowest alkali content (0.53%), which can be considered a low-alkali cement. This parameter plays a key role in the alkali–silica reaction (ASR) [20] as well as in the mechanical strength development and flowability of mortars and concretes. Given this, the condition for mortar to conduct the alkali–silica reaction (ASR) is that the cement, mixing water or additions, among other sources, must contain alkali. 

As mentioned above, the alkali content of coal fly ashes for inclusion in mortars is important. The use of low alkali coal fly ash can aid in suppressing the alkali–silica reaction (ASR) because it results in alkali dilution. Furthermore, the slower pozzolanic reaction seems to cause lower hydroxyl ion concentrations and denser microstructures. Both effects act in a positive manner on the mitigation of the alkali–silica reaction. Some applications in which coal fly ash is used set an upper limit for this addition of 2.0% (Na_2_O_eq_).

Pozzolanic materials lower the CaO/SiO_2_ ratio of C-S-H gel formed during the pozzolanic reaction, which allows more alkalis to be incorporated into its structure. Around 95 per cent of the total alkali content could be immobilized in calcium silicate hydrates by blended cement pastes, compared to about 15 percent in those containing only Portland cement. In solid phases, potassium is present in higher concentrations than sodium [24].

### 3.3. Compressive Strength

**Figure 4:**
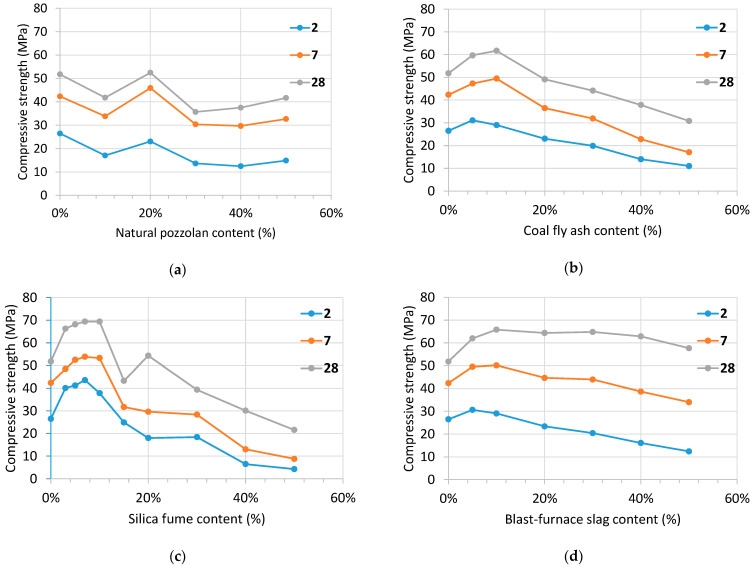
Compressive strength at 2, 7 and 28 days of the mortars made with the reactive siliceous pebbles and the four additions mixed in several percentages: (**a**) natural pozzolan, P; (**b**) siliceous coal fly ash, V; (**c**) silica fume, D; (**d**) blast-furnace slag, S.

Figure 4 shows the compressive strength at 2, 7 and 28 days of the mortars made with the reactive siliceous pebbles and the four additions (natural pozzolan, P, siliceous coal fly ash, V, silica fume, D, and blast-furnace slag, S) mixed in several percentages.

Compressive strength’s silica fume mortars increase up to 7–10% of content for all ages. Then, it dramatically decreases because the amount of Ca(OH)_2_, produced in the Portland cement hydration reaction, which is available to react with the reactive silica is over. By contrast, the natural pozzolan and blast-furnace slag showed quite a different performance because their silica content is much smaller, i.e., 43% and 35%, respectively. Therefore, both of them are able to combine calcium hydroxide by using a higher amount of pozzolanic material. The amount of SiO_2_ in the siliceous coal fly ash is about 52%. This fact justifies intermediate performance with regard to the other additions.

**Figure 5:**
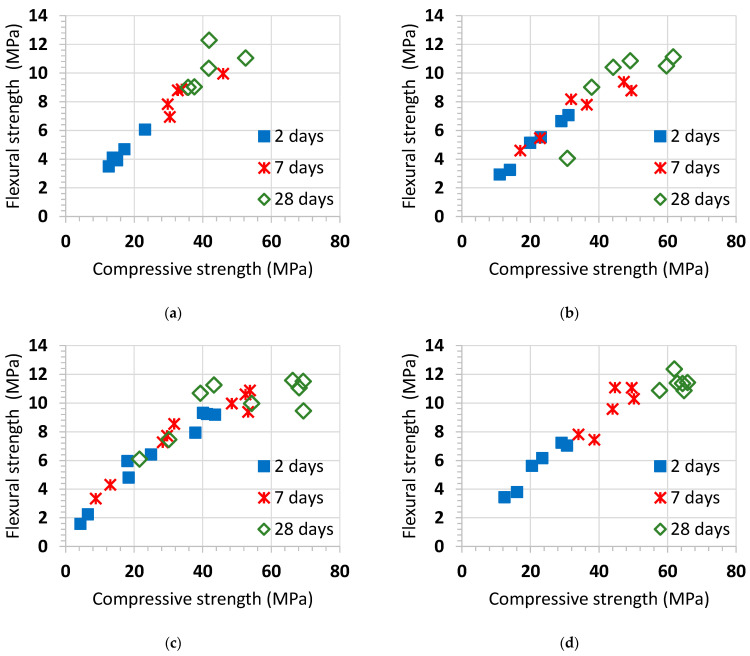
Relationship between compressive strength and flexural strength results for the mortars made with the reactive siliceous pebbles and the four additions mixed in several percentages: (**a**) natural pozzolan, P; (**b**) siliceous coal fly ash, V; (**c**) silica fume, D; (**d**) blast-furnace slag, S.

Figure 5 represents the relationship between compressive strength and flexural strength results of the mortars made with the reactive siliceous pebbles and the four additions (natural pozzolan, P, siliceous coal fly ash, V, silica fume, D, and blast-furnace slag, S) mixed in several percentages.

Flexural strength increases when the compressive strength and age of the mortar increase. Nevertheless, the increase in flexural strength is lower than the corresponding increase in compressive strength at same age of mortar. In particular, silica fume mortars exhibit a curved correlation between the compressive strength and the flexural strength at 28 days. 

Correlations of flexural and compressive test results have been determined, but it is only an approximation. However, in some practical cases, this is a good way to reinforce knowledge of the material [25]. An almost linear relationship between flexural and compressive test results was found at early ages, but it was broken at 28 days, probably due to the different strength gain promoted by the four pozzolanic additions. Pozzolanic reaction and beginning of the alkali–silica reaction (ASR) is different for each material.

### 3.4. Expansion

**Figure 6:**
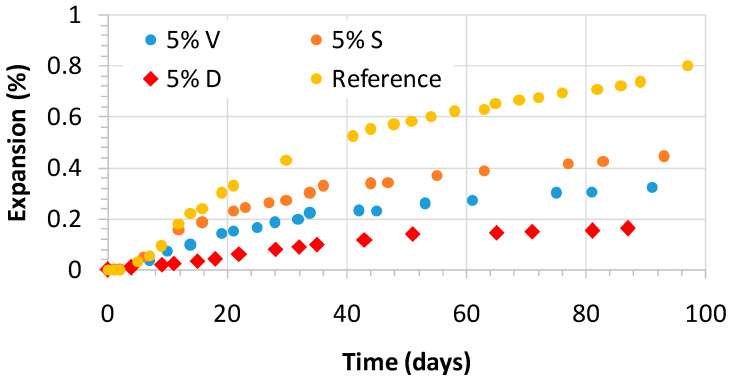
Alkali–silica mitigation by the siliceous coal fly ash, V; silica fume, D; blast-furnace slag, S, mixed in the mortars made with the reactive sand in comparison with the mortar elaborated with the reference cement (CEM I).

Figure 6 displays the alkali-silica mitigation by the siliceous coal fly ash, V; silica fume, D; ground granulated blast-furnace slag, S, mixed in the mortars made with the reactive sand in comparison with the mortar elaborated with the reference cement (CEM I). The expansion provoked by the alkali–silica reaction (ASR) appears to depend on the basicity of the binder matrix, with a higher basicity leading to a greater level of expansion. Accordingly, the use of the four pozzolanic materials in the Portland cement lowers the lime content and mainly increases the SiO_2_ content, and sometimes the Al_2_O_3_ and Fe_2_O_3_ contents of the final pozzolanic cement, therefore reducing its basicity [26]. This fact justifies why pozzolanic additions with a higher CaO content are normally less effective in mitigating ASR expansion in accordance with the results found in the literature [27,28]. 

According to Ramjan et al. [29], the extent of the alkali–silica reaction (ASR) mitigation depends on the replacement level by coal fly ash rather than on its fineness. They suggest that reducing the CaO content in mortars is more effective in controlling the alkali–silica reaction (ASR) expansion than the filler effect promoted by the Portland cement replacement. Given that, the basicity of the blended cement matrix can be checked by the (CaO)/(SiO_2_ + Al_2_O_3_ + Fe_2_O_3_) ratio in order to assess the effectiveness of controlling expansion (Table 4).

The linear relationship shown in Equation (3) has been obtained between the expansion at 90-days of testing for mortars with a 5% of replacement of siliceous coal fly ash, V; silica fume, D; blast-furnace slag, S, with a coefficient of determination r^2^ of 0.94, which is good. In general, the higher the r^2^, the better the equation fits the data. Greater input data would likely improve the model. Nevertheless, this equation indicates that the CaO content of the binder plays a key role regarding the alkali–silica reaction (ASR) expansion.

Expansion (%) = 4.2199 [(CaO)/(SiO_2_ + Al_2_O_3_ + Fe_2_O_3_) ratio] − 0.947 (3)

**Figure 7:**
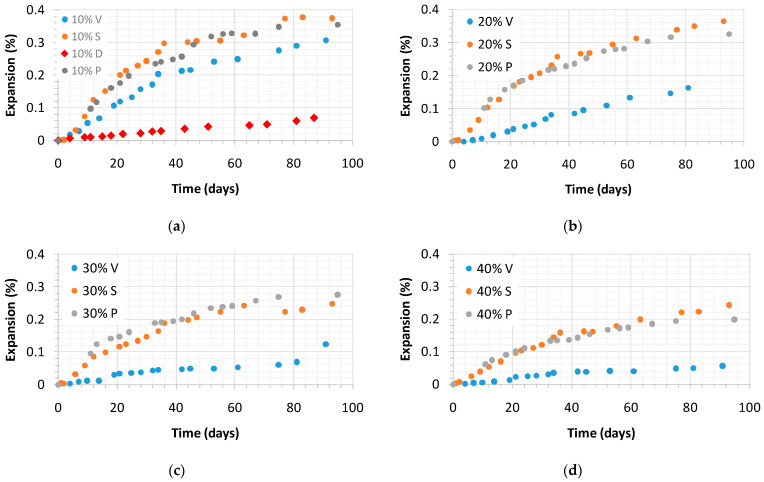
Alkali–silica mitigation by the four pozzolanic additions (natural pozzolan, P; siliceous coal fly ash, V; silica fume, D; blast-furnace slag, S, mixed in the mortars made with the reactive sand in four percentages: (**a**) 10%; (**b**) 20%; (**c**) 30%; (**d**) 40%.

According to what is stated above, Figure 6 and Figure 7 reflect a significant mitigation of the alkali–silica reaction (ASR) expansion obtained by decreasing the CEM I content in the mortar mixes through the partial replacement with the four pozzolanic materials. Figure 6 compares the expansion presented by the mortar elaborated with the reference cement (CEM I) with mortars made with 5% of pozzolanic additions.

It is remarkable, however, how small a proportion of the pozzolanic addition (5%) subsequently led to expansion control. Silica fume is the most effective, followed by coal fly ash and ground granulated blast-furnace slag. Nevertheless, the higher the addition content in the mortar, the lower the expansion that was observed, as shown in Figure 6. This trend is clearer in mortars with siliceous coal fly ash, V, compared to the case of mortars with natural pozzolan, P, or ground granulated blast-furnace slag, S. In cases where P or S are used, higher amounts (30% or 40%) are needed for effective alkali–silica reaction (ASR) mitigation. 

The highest Na_2_O_eq_ content found in the coal fly ash was 4.56%, and for silica fume this was 3.22%. On the other hand, Portland cement is considered a low-alkali cement (0.53%). The mentioned high alkali levels in the additions do not negatively affect alkali–silica reaction (ASR) expansion. To an even lesser extent in mortars made with coal fly ash, alkaline aluminosilicate gel is formed as the result of a very complex chemical process. Accordingly, the slow pozzolanic reaction might be enhanced and a denser microstructure obtained [20].

The typical expansion curve of a mortar or concrete affected by alkali–silica reaction (ASR) is characterized by three zones: (i) beginning of the swelling; (ii) substantial expansion at constant rate, and (iii) the final stage is marked by slowly decreasing the expansion rate to reach a plateau. However, in the expansion curves plotted in Figure 6 and Figure 7, the first and second zones are not clearly separated. Furthermore, contrary to what could have been expected [30,31,32,33,34], there was no plateau at the end of the curves, i.e., there is a final slope that is different from zero. This is possibly because cement replacement slowed down the ASR reaction; accordingly the curve was not yet flat.

Some authors attribute this final slope to the expansion of marginally reactive aggregates initially classified as non-reactive [35], but most of the authors do not consider this final expansion because it is not obvious. This performance is due to the swelling development in accelerated tests that have been going on for several years. In addition, some chemical or physical causes lead to dimensional changes that can affect the final expansion and are not related to the alkali–silica reaction (AAR). For instance, such changes can be promoted by the pozzolanic reaction, delayed ettringite formation, storage conditions (temperature and relative humidity), and so on.

### 3.5. Microanalysis of Pozzolanic Cement Mortars

**Figure 8:**
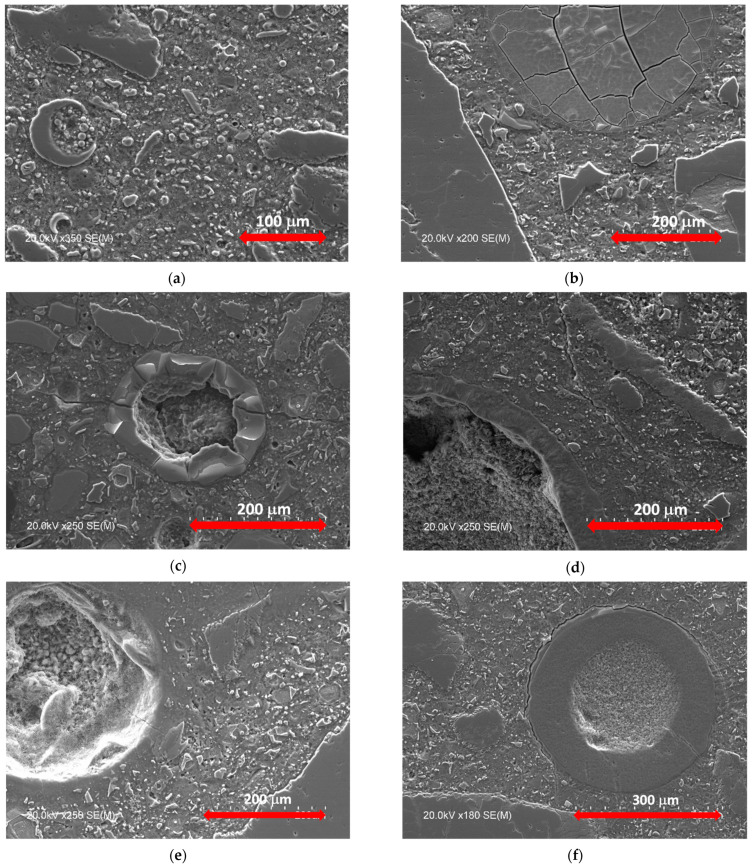
Backscattered SEM images of mortars made with the reactive aggregate following 90 days of accelerated testing and further storage for 30 months in a 1N NaOH solution: (**a**) mortar with 20% of siliceous coal fly ash; (**b**) mortar with 20% of blast-furnace slag; (**c**) mortar with 20% of silica fume; (**d**) mortar with 20% of natural pozzolan; (**e**) mortar without additions (ASR gel filling partially a pore and microcracking); (**f**) mortar without additions showing ASR gel in a pore.

In the aftermath of the 90 days of accelerated mortar bar expansion testing, prism specimens used in the tests were stored in containers submerged in a 1 N NaOH solution for 30 months. Soon after, mortars were prepared for scanning electron microscopy (SEM) examination using backscattered electron imaging contrast (Figure 8). The mortar control sample presented outstretched cracking, whereas blended cement mortars showed lower cracking or a compact aspect without cracks. Cracks were found within the reactive aggregates or at the interface paste-aggregate. Some of them were partially filled with ASR gel.

Figure 8 shows the backscattered SEM images of mortars made with the reactive aggregate and 20% of the four additions considered in the present study, after 90 days of accelerated testing and further storage for 30 months in a 1N NaOH solution. Some pores were filled partially or totally with a mixture of the reaction products formed by the ASR gel. This observation was mainly found in the mortars made with ground granulated blast-furnace slag; natural pozzolan and silica fume. However, mortars made with siliceous coal fly ash there were not any visually apparent crack due to the alkali–silica reaction (ASR), which is in concordance with the low expansion results of this type of mortar (Figure 7).

The microstructural observations by backscattered SEM images suggest that percentages of replacement of 20% partially control the alkali–silica reaction (ASR) in the case of ground and granulated blast-furnace slag; natural pozzolan and silica fume mortars. Only emerging microstructural changes, due to the alkali–silica reaction (ASR), were evidenced.

## 4. Conclusions

In this study, the influence of several percentages of natural pozzolan, P, siliceous coal fly ash, V, silica fume, D, or ground granulated blast-furnace slag, S, on the open porosity, mechanical strength, and alkali–silica reaction (ASR), of blended cement mortars were analyzed and discussed. The following conclusions were drawn:

1. The reference mortar without additions presented an open porosity of 15.25%. Mortars made with natural pozzolan, P, siliceous coal fly ash, V, or ground granulated blast-furnace slag, S, showed lower open porosities for all the replacement levels. This finding was the same for mortars made with low contents of silica fume (3% and 5% D). As a pozzolanic materials, they react with the calcium hydroxide formed by the calcium silicate hydration to form C-S-H gel with a lower Ca/Si ratio, making the mortar mixes more compact and homogeneous.
2. Linear relationships between compressive and flexural strength were found for natural pozzolan, P, siliceous coal fly ash, V, and ground granulated blast-furnace slag, S. By contrast, silica fume mortars only showed such linear relationship at 2 and 7 days. At 28 days, a maximum flexural strength of about 11 MPa was reached, while compressive strength ranged from 40 to 70 MPa.
3. Low replacements with silica fume (from 7% to 10% D) are enough to prevent alkali-silica reaction (ASR). The second-best addition to control ASR is the siliceous coal fly ash with replacement levels above 20%. In contrast, natural pozzolan, P, and ground granulated blast-furnace slag, S, showed similar performances. They are effective, starting at 30%. It is suggested that a reduction in the CaO content in mortars is effective in controlling the alkali–silica reaction (ASR) expansion.
4. The alkali–silica reaction (ASR) expansion curves were characterized by three zones: (i) beginning of the swelling; (ii) substantial expansion at constant rate; (iii) final stage showing a slope different from zero. This could be explained by the expansion of marginally reactive aggregates, the swelling that continues for several years in accelerated tests, and some dimensional changes due to chemical or physical factors.
5. Finally, the reference mortar prism presented widespread cracking, while blended cement mortars depict smaller or non-cracking with a denser microstructure.

## Figures and Tables

**Table 1:** Percentage of the pozzolanic additions in the blended cement for ASR testing.

Pozzolanic addition	Percentage of the Additions in the Blended Cement				
Silica fume, D	5%	10%	-	-	-
Siliceous coal fly ash, V	5%	10%	20%	30%	40%
Ground granulated blast-furnace slag, S	5%	10%	20%	30%	40%
Natural pozzolan, P	-	10%	20%	30%	40%

**Table 2:** Percent chemical composition by mass of the Portland cement, CEM I, natural pozzolan, P, siliceous coal fly ash, V, silica fume, D, ground granulated blast-furnace slag, S, and reactive aggregate.

Parameter	CEM I 42.5 N	V	S	D	P	Aggregate
Al_2_O_3_	3.65	23.16	12.16	-	13.15	15.80
CaO	64.49	5.75	41.55	0.60	12.98	4.30
Cl^−^	0.03	-	-	0.06	-	0.02
Cr_2_O_3_	0.02	0.17	-	-	-	0.04
Fe_2_O_3_	3.96	5.93	0.39	0.14	12.75	6.83
K_2_O	0.64	0.96	0.48	3.03	0.56	4.58
MgO	1.27	1.96	6.96	0.33	9.62	2.47
Mn_2_O_3_	0.05	0.06	0.22	-	-	0.08
Na_2_O	0.12	3.93	0.17	1.23	0.63	2.88
P_2_O_5_	0.18	0.67	-	-	1.26	0.45
SiO_2_	20.80	52.17	35.13	91.31	42.82	60.55
SO_3_	2.33	0.36	1.86	-	0.00	0.02
SrO	0.06	0.10	0.05	-	0.12	0.03
TiO_2_	0.19	0.92	0.56	-	3.62	1.00
ZnO	0.01	0.02	-	-	0.02	-
ZrO_2_	-	0.03	0.02	-	0.05	0.03
LOI ^1^	2.21	3.80	0.45	3.29	2.21	0.81
Na_2_O_eq_ ^1^	0.54	4.56	0.49	3.22	0.99	5.89

^1^ LOI: Loss on ignition; Na_2_O_eq_ = Na_2_O + 0.658 K_2_O.

**Table 3:** Alkali content in the mortars made with blended cements.

%Na_2_O_eq_ ^1^	Pozzolanic Material Content in the Binder (%)									
	0%	3%	5%	7%	10%	15%	20%	30%	40%	50%
Silica fume, D	0.53	0.61	0.66	0.72	0.80	0.75	1.07	1.34	1.61	1.88
Coal fly ash, V	0.53	-	0.73	-	0.93	-	1.34	1.74	2.14	2.55
Ground granulated blast-furnace slag, S	0.53	-	0.53	-	0.53	-	0.52	-	0.51	0.51
Natural pozzolan, P	0.53	-	-	-	0.58	-	0.62	0.67	0.72	0.77

^1^ Na_2_O_eq_ = Na_2_O + 0.658 K_2_O.

**Table 4:** Basicity of the binder materials measured by the (CaO)/(SiO_2_ + Al_2_O_3_ + Fe_2_O_3_) ratio.

Material	CEM I 42.5 N	V	S	D	P	Aggregate
(CaO)/(SiO_2_ + Al_2_O_3_ + Fe_2_O_3_)	2.27	0.07	0.87	0.01	0.19	0.052

## Data Availability

The data presented in this study are available on request from the corresponding author.
